# Development of a SNP barcode to genotype *Babesia microti* infections

**DOI:** 10.1371/journal.pntd.0007194

**Published:** 2019-03-25

**Authors:** Mary Lynn Baniecki, Jade Moon, Kian Sani, Jacob E. Lemieux, Stephen F. Schaffner, Pardis C. Sabeti

**Affiliations:** 1 Broad Institute, Cambridge, Massachusetts, United States of America; 2 Department of Organismic and Evolutionary Biology, Harvard University, Cambridge, Massachusetts, United States of America; 3 Division of Infectious Disease, Massachusetts General Hospital, Boston, Massachusetts, United States of America; 4 Department of Immunology and Infectious Diseases, Harvard School of Public, United States of America; Johns Hopkins Bloomberg School of Public Health, UNITED STATES

## Abstract

*Babesia microti* is tick-borne disease that is an emerging threat to public health due to increasing prevalence and expanding geographic range. Detection and constant surveillance of babesiosis is imperative for predicting pathogen expansion. Leveraging our whole genome sequence (WGS) analyses of *B*. *microti*, we developed a single nucleotide polymorphism (SNP)-based high resolution melt (HRM) surveillance tool. We developed our HRM assay using available sequence data and identified 775 SNPs. From these candidate SNPs, we developed a 32-SNP barcode that is robust and differentiates geographically distinct populations; it contains SNPs that are putatively neutral, located in nuclear, mitochondrial, and apicoplastal regions. The assays are reproducible and robust, requiring a small quantity of DNA (limit of detection as low as 10 pg.). We analyzed the performance of our HRM assay using 26 *B*. *microti* clinical samples used in our WGS study from babesiosis endemic regions in the United States. We identified a minimal barcode consisting of 25 SNPs that differentiate geographically distinct populations across all clinical samples evaluated (average minor allele frequency > 0.22). Supporting our previous WGS findings, our 25-SNP barcode identified distinct barcode signatures that segregate *B*. *microti* into two lineages: Northeast and Midwest, with the Northeast having three subpopulations: Connecticut/Rhode Island, Nantucket, and the R1 reference group. Our 25-SNP HRM barcode provides a robust means genetic marker set that will aid in tracking the increasing incidence and expanding geographic range of *B*. *microti* infections.

## Author summary

*Babesia microti* is an emerging tick-borne disease and is becoming a public health problem. Over the past two decades, the *I*. *scapularis* tick population, which is primarily responsible for human infection, has exploded, doubling the number of babesiosis and other *I*. *scapularis-*borne disease cases to approximately 48 thousand reported in 2016. The increasing number of endemic areas of *B*. *microti* signals the need to develop robust and accurate surveillance tools to effectively monitor and record disease incidence. Here, we used our whole genome sequence analysis of *B*. *microti* to develop a genetic barcode for *B*. *microti*, composed of 25 robust variants. We show the validation and utility of this SNP barcode on 26 babesiosis positive clinical samples from endemic regions in the United States. This genetic barcode provides a means to identify the genetic origin of a parasite and ultimately gain insight into *B*. *microti* population structure and transmission dynamics.

## Introduction

Human babesiosis is an increasingly recognized tick-borne disease with the vast majority of cases in the United States caused by the malaria-like protozoan *Babesia microti [1, 2].* Infections occur predominantly through the bite of an infected *Ixodes scapularis* tick but can also occur via the transfusion of blood products [3, 4]. Human babesiosis, like malaria, is characterized by fever and haemolysis [3]. Severity of infection ranges from asymptomatic to severe and can be complicated by hemolytic anemia, and respiratory or organ failure [5].

Since the first reported case of human babesiosis in the United States on Nantucket in 1969, the geographic range and incidence of babesiosis has increased [3, 6–8]. Human infections primarily occur in the United States (U.S.), particularly in New England, New York, and North Central Midwest [2–5, 6, 7]. This led the Center for Disease Control and Prevention (CDC) in 2011 to classify human babesiosis as an emerging and nationally notifiable disease [9]. In 2014, the CDC reported babesiosis cases rose from 1,126 to 1,744 with 94% of the cases in Connecticut, Massachusetts, Minnesota, New Jersey, New York, Rhode Island and Wisconsin (https://www.cdc.gov/parasites/babesiosis/data-statistics/index.html).

The increasing number of endemic areas of *B*. *microti* signals the need to effectively track and record disease incidence. In this study, we leveraged our whole genome sequence (WGS) analyses of *B*. *microti* [1] and developed a High Resolution Melt (HRM) [10] single nucleotide polymorphism (SNP) genotyping method. Previously, we have developed SNP barcodes for to both *Plasmodium vivax* and *P*. *falciparum* and have employed them to successfully monitor malaria transmission using HRM analysis [11, 12]. Leveraging our experience, we designed a 32-SNP barcode for *B*. *microti* that contains SNPs that are located in nuclear, mitochondrial, and apicoplastal regions. We further reduced this set to minimal 25-SNP barcode for *B*. *microti* that can uniquely identify the geographic origin of the sample and may ultimately provide genomic insight for various population parameters. The 25-SNP barcode was evaluated in a pilot screen of 26 clinical samples from babesiosis endemic regions in the continental U.S.: Mainland New England (MNE—Massachusetts, Maine, Connecticut, Rhode Island and New Hampshire), Midwest (MW—Wisconsin, Minnesota, and North Dakota) and Cape Cod (NAN—Nantucket). The development of this 25-SNP barcode for *B*. *microti* serves to enhance epidemiological surveillance of this emerging pathogen.

## Materials and methods

### *Babesia microti* genomic DNA samples

We used *B*. *microti* strains that were sequenced at the Broad Institute as internal controls to identify both reference and alternate alleles for each assay [1]. The reference alleles were based on version 1 of the sequencing information available in Genbank [13]. The original R1 reference (REF) contained three chromosomes, where chromosome 3 was a supercontig (GI: 399217317). In the revised version, the supercontig was broken to two chromosomes, chromosomes 3 (GI: 908660426) and 4 (GI: 908661396), for a total of four nuclear chromosomes. For barcode development, we used version 1 for the original R1 reference. Every SNP in our barcode can be mapped to a position in the revised model. We used 26 clinical samples for validation of our *B*. *microti* barcode assays. As previously described by Lemieux and colleagues [1], study participants included patients with a positive *Babesia* smear or *B*. *microti* PCR test. The samples were collected at study sites in four regions in the continental United States: MNE [Massachusetts (8 samples), Maine (1), Connecticut (1), and New Hampshire (1)], MW (Wisconsin (1), Minnesota (2) and North Dakota (1)] and NAN [Nantucket (7)] and unknown origin (4). Venous blood draws of 5–10ml was collected in a heparin tube at the time of enrollment and stored at 4°C for less than 3 weeks.

### Ethics statement

We obtained clinical samples through the Massachusetts General Hospital (MGH) microbiology laboratory, Brigham and Women's Hospital (BWH), and at the University of Massachusetts Medical School (UMMS) hospital clinical laboratory at UMMS. As previously described by Lemieux and colleagues [1], informed consent was obtained for all study participants at MGH and BWH according to Partners Institution Review Board (IRB) protocol 2014P000948. Samples obtained from the UMMS were IRB-exempted de-identified discarded peripheral blood samples. We obtained historical samples from The Mayo Clinic as discarded, de-identified specimens. Finally, we obtained historical laboratory-adapted strains and tick samples from Tufts School of Veterinary Medicine, including serial isolates of the GI strain of a recurrent *I*. *scapularis* (tick) and rodent passage from 1986 to 2014 and the RMNS strain.

### DNA sample preparation

Genomic DNA was previously harvested from whole blood samples from babesiosis positive (i.e, diagnosed by BSA) patients in heparinized tubes. Next, the genomic DNA was extracted from the samples using the *QIAamp DNA Blood Mini Kit* (QIAGEN). The concentration of DNA for all clinical samples was quantified using the *Qubit 3*.*0 Fluorometer* (Invitrogen). Next, whole genome amplification (WGA) was performed on the clinical samples using the illustra Ready-To-Go Whole GenomiPhi V3 DNA Amplification Kit (GE Healthcare Life Sciences) according to manufacturer’s instructions. For each WGA reaction, 2 μl of each clinical sample was used to yield 40 μl amplified product. Following WGA, the samples’ DNA concentration was quantified using the Qubit 3.0 Fluorometer.

### Development of qPCR primers, probe and assay conditions

We developed a specific *B*. *microti* qPCR assay for research use to detect and quantitate our babesiosis samples. The *B*. *microti qPCR* assay targets the cytochrome oxidase B, CytB gene (NCBI ID: 13435122). We scanned the *B*. *microti* genome (NCBI ID: 11700) using Geneious software (version 6.1) (Biomatters Ltd.). We identified a 118 base pair region in the CytB gene that was highly conserved. We used BLAST genomic database search (National Library of Medicine (NLM) to confirm that the region was specific to known pathogen *microti* strains (RI, Gray and MN-1). We designed primers and probes using Primer3 [14] and Realtime PCR Tool (Integrated DNA Technologies, IDT). We checked primer pairs for potential primer dimers and their specificity using Geneious software (version 6.1), and we used BLAST to screen for species specificity. We identified optimized primer sequences for the forward primer as CCTAGGTATGTATCATCTTAACCTCTTT and the reverse primer sequence as TAGGGATCGTAGTCGTGTACTG. We used PrimeTime Probes (Integrated DNA Technologies, IDT) with the fluorescent dye FAM on the 5’end is double-quenched in the center by ZEN and by on its 3’end of the probe sequence (CCCAAGTAGGTATCTATGTACTTCTACTGT). We performed the qPCR assay using the LightCycler 96 System (Roche). The reaction consisted of 5.0 μl LightCycler FastStart Essential DNA Probes Master Mix (Roche), 2.0 μl of 0.1 μM forward and reverse primer mixture, (2.0 μl) 0.1 μM probe, and 1 μl of DNA sample in a total reaction volume of 10 μl. The 2-step amplification cycling conditions were as follows: 95°C for 10 min and 40 cycles at 95°C for 15 secs, and 60°C for 60 secs.

### Limit of detection of the *B*. *microti* qPCR

We determined the limit of detection (LoD) for the qPCR assay by selecting the lowest gDNA concentration which was able to reproducibly, in triplicate, yield a cycle threshold ≤ 36 cycles. We then ran 20 reactions with template gDNA at that observed LoD. We defined the LoD criteria as having a confidence interval (CI) of ≥ 90%. If these criteria were not met, we would repeat the assay at a higher concentration.

### Identification of candidate HRM barcode SNPS

We identified candidate SNPs for our *B*. *microti* barcode by using the UnifiedGenotyper tool from the Genome Analysis ToolKit (GATK) [15] with minimum quality score of 50 [1]. We further filtered the identified SNPs by performing principal component analysis (PCA) using the R package ‘*stats’ [16].* We selected SNPs with a PCA component absolute score (absolute value of the transformed variable values corresponding to a particular data point) greater than 3, 2, then 0.5. The principal components score represents the distance from the origin in the transformed space, so the greater the absolute value of the principal components score, the more informative the SNP is at that position. We then screened these candidate SNPs by selecting for putatively neutral mutations (intergenic, intronic, and 4-fold degenerate sites) and for mutations that resulted class I and II SNPs mutations (A>G, A>C, T>G, T>C, G>A, G>T, C>A, C>T). Finally, we used PCA to identify SNPs that visually create geographic clustering. We assessed the significance of PCA results graphically and by using percent of variance (POV), which is calculated as the sum of eigenvalues corresponding to the first and second principal components over the sum of all eigenvalues [17].

### Primer design and assay selection for SNP-Barcode

We designed primer pairs using the LightScanner primer design software version 2.0 (BioFire Defense, USA). The software uses standard design parameters for HRM, such as primer length (18–28 nucleotides), melting temperature (58–60°C), GC content (40–60%) and amplicon size (<60 bp) to design optimal primer pairs for each SNP. We then tested the designed primer pairs *in silico* using uMelt [18], a flexible web-based tool for predicting DNA melting curves and denaturation profiles of PCR products. Based on the results derived from uMelt, we selected assays with a temperature melt (Tm) separation of ≥ 0.8°C between the reference and the alternate SNP allele. To ensure the specificity of our primers, we ran a BLAST genomic database search (National Library of Medicine (NLM), USA) to compare each primer and probe sequence against common pathogens.

### HRM method and optimization for SNP-Barcode

We identified the optimal PCR profile in clinical samples as a two-step protocol. The PCR profile involved 120 sec at 95°C; 40 cycles of 30 sec at 95°C and 60 sec at 60°C; and a final HRM cycle of 15 sec at 55°C and 15 sec at 95°C. The optimized master mix for the PCR 12 μl reaction contained 1 μl of DNA sample containing 10 ng/μl DNA in 1X TE Buffer, 1.2 μL of PCR grade water (VWR, Radnor, PA, USA), 4.8 μL of 2.5X LightScanner master mix (BioFire Diagnostics Inc., Salt Lake City, Utah, USA), and 5 μL of primer solution containing 0.1 to 0.5 μM of forward and 0.1 to 0.5 μM reverse primers diluted in 1X TE Buffer depending on the individual assay (Integrated DNA Technologies) for a total reaction volume of 12 μL. All assays were performed on an Applied Biosystems ViiA 7 Real-Time PCR System or QuantStudio 6 (Life Technologies). To optimize the HRM assays, we evaluated five different primer concentrations (0.1 μM, 0.2 μM, 0.3 μM, 0.4 μM, and 0.5 μM) while leaving all other reaction conditions unchanged. We selected primer concentrations based on the correct amplicon size, the cycle threshold (C_T_) ≤ 30, having a single melt profile, and being robust with an efficiency between 90–100%. We defined a successful assay as having a C_T_ ≤ 30, because cycle thresholds greater than 30 can result in shifted melt profiles, resulting in unreliable genotyping data [10]. We used the HRM method to genotype our SNPs following the defined protocol. To perform the HRM assay, we first quantified the concentration of DNA for all clinical samples, as described above using a Nanodrop. We then diluted all clinical samples using 1x TE Buffer to 10 ng/μl based on OD_260._ For all assays, we included sequenced control samples to identify the reference and alternate allele SNP temperature melt (Tm) curves. Next, we prepared a reaction master mix, consisting of 1.2 μL of PCR grade water (VWR, Radnor, PA, USA), 4.8 μL of 2.5X LightScanner master mix (BioFire Diagnostics Inc., Salt Lake City, Utah, USA), and 5 μL of primer solution containing 0.1 to 0.5 μM of forward and 0.1 to 0.5 μM reverse primers diluted in 1X TE Buffer depending on the individual assay (Integrated DNA Technologies, Inc.). We added 1 μl of diluted DNA sample (10 ng/μl) to this master mix. Once we prepared our plate, we gently centrifuged them before beginning PCR, using the profile described above.

### Assay efficiency and sensitivity for *B*. *microti* qPCR and SNP-Barcode

We evaluated assay efficiency using the standard curve method for qPCR. We prepared the DNA sample, that was previously amplified using WGA, by using six 10-fold serial dilutions (10^3^ ng/μl to 1 ng/μl) of our clinical control sample (Gray) and Reference 1 (REF). We generated the standard curve from the qPCR assay to calculate the percent efficiency of each assay and to determine their sensitivity or the minimum amount of DNA needed for successful amplification.

### *In silico* cross reactivity evaluation of the *B*. *microti* and SNP-Barcode assays

We evaluated the *B*. *microti* qPCR and SNP barcode assays for analytical specificity *in silico* by comparing each primer and probe sequence against those of other common causes of pathogenic illness in humans including other protozoan species including: *Plasmodium (P*. *falciparum*, *P*. *vivax*, *P*. *knowlesi*, *P*. *malariae*, *P*. *ovale)*, *Babesia (B*. *divergens*, *B*. *divergens-like*, *B*. *venatorum*, *B*. *rodhaini*, *B*. *bovis)*, *and Theileria parva*. We used BLAST to evaluate all primers and probes for cross species reactivity using all possible combinations.

### Genotyping reproducibility using the SNP-Barcode

We tested control samples for each assay and used the derivative Tm curve to identify the reference and alternate alleles for each SNP assay. We evaluated genotyping reproducibility by performing each SNP assay in duplicate. We then calculated the mean Tm differences and standard error between duplicates to evaluate reproducibility and robustness of each assay. Additionally, we further tested genotyping accuracy of our barcode assays on 26 clinical samples. For these clinical samples, we compared HRM SNP data to WGS SNP calling to reaffirm the genotyping reproducibility of our method.

### Genomic analysis using SNP-Barcode

We calculated the minor allele frequency (MAF) from allele counts for each SNP in each population. We assessed sample uniqueness by comparing genotypes across all pairs of samples. We classified pairs of distinct monomorphic genotypes (e.g. A/G). We performed PCA using the program SmartPCA in the Eigensoft package [17,19] on the diverse panel of 26 *B*. *microti* clinical samples. We calculated the POV explained as the sum of eigenvalues corresponding to the first and second principal components over the sum of all eigenvalues.

## Results

### *B*. *microti* qPCR assay is robust and reproducible

We evaluated the robustness and efficiency of the *B*. *microti* qPCR assay by performing the standard curve method using a 10-fold dilution series of the DNA. We performed this experiment in triplicate and had a resultant efficiency of 99.4% (R2 = 0.99). Based on the standard curve method, we identified the tentative LoD as 3 pg. We confirmed the LoD by testing 20 replicates at 3 pg and found 19 out of 20 samples were detected (CI = 95%) with an average PCR cycle threshold (C_T_) of 34.68. We used BLAST to evaluate assay specificity and potential cross-reacting target sequences and did not identify any significant sequence matches. We evaluated reproducibility and performance of the *B*. *microti* qPCR assay by performing a pilot screen in duplicate using our 26 clinical samples, that were previously amplified by WGA, from MNE, MW and Cape Cod that were previously confirmed positive in our WGS study [1] and one sample of *Lyme borreliosis* as a negative control. We identified 26 out of 26 *B*. *microti* samples with an average standard of error of ± 0.16 and found that the *L*. *borreliosis* was negative with no amplification (S1 Table).

### Sequence analysis and HRM assay optimization identifies a *B*. *microti* 32-SNP barcode

Our selection criteria to identify candidate SNPs for the *B*. *microti* SNP barcode included using putatively neutral genomic loci (intergenic, intronic, or 4-fold degenerate sites) located in nuclear, mitochondrial, and apicoplastal regions. Using *B*. *microti* genome information from our previous sequencing efforts [1] on 32 human babesiosis clinical samples from the Northeast (NE) (MNE, Sandy Neck, and NAN) and MW (Minnesota and Wisconsin), we identified 2,445 candidate SNPs. We screened these candidates using PCA and selected 775 SNPs that captured a high degree of population diversity and that differentiated geographically distinct populations. Further screening these candidates for putatively neutral genomic loci and for class I and class II mutations, which have the broadest SNP melt temperature (Tm) separation (A>G, A>C, T>G, T>C, G>A, G>T, C>A, C>T), we identified a final set of 477 candidate SNPs for HRM assay development.

Next, we winnowed these candidates down based on HRM primer design guidelines. We used uMelt [18], a flexible web-based tool for predicting DNA melting curves and denaturation profiles of PCR products, and selected 91 candidates SNPs that had the strongest performing assays *in silco*. Targeting these 91 SNPs, we designed primers for HRM analysis that were specific to *B*. *microti* (S2 Table). We tested these HRM assays for species specificity and the ability to distinguish between alternate and reference alleles, with a temperature separation greater than 0.8°C. This yielded a 32-SNP barcode, containing putatively neutral genomic loci and including 17 nuclear, 14 mitochondrial, and 1 apicoplastal SNPs (S3 Table).

### *B*. *microti* HRM assays are robust and reproducible

We evaluated the efficiency and LoD of the assays by performing the standard curve method using 10-fold serial (10^3^ ng/μl to 1 ng/μl) dilutions in triplicate. The efficiency of our assays ranged from 90–100%, and the LoD varied from 1 to 10 pg (S4 Table). We evaluated the reproducibility and performance of our 32-SNP barcode by screening the 26 clinical samples, that were previously amplified by WGA, used to test our *B*. *microti* qPCR assay and one *L*. *borreliosis* sample, used as a negative control. We ran the pilot screen using the Applied Biosystems ViiA 7 Real-Time PCR System. The assays were highly sensitive and accurate, correctly genotyping all 26 clinical samples (832 out of 832 SNP calls and concordant across all duplicates). The *L*. *borreliosis* sample was negative with no amplification. The assays were robust, with the variability in the T_m_ values for each assay run in duplicate of < 0.12°C for all 32 assays and the average standard error ranging from ≥ ± 0.02 to ≤ ± 0.20°C (S5 Table).

### A 25-SNP barcode that differentiates population diversity

Together, this set of 32 SNPs spans all three nuclear chromosomes, mitochondrial and apicoplastal regions, with the closest pair of nuclear SNPs at least 801 bp apart. On average, nuclear SNP pairs are 166,646 bp apart, mitochondrial SNPs are 101 bp apart, and apicoplastal SNPs are 2023 bp apart. Together, this set of 32 SNPs successfully distinguished babesia samples tested from populations in MNE, the MW, and NAN. The barcode distinguished all samples according to geographic origin, except for one sample from North Dakota (ND11) which grouped with the MNE samples, and one sample from South Dennis, MA (Bab14) which grouped with the NAN samples (S6 Table). The SNPs captured high degrees of diversity with the average MAF value > 0.22 for 53% of the SNPs (17 out of 32) (S7 Table).

**Fig 1:**
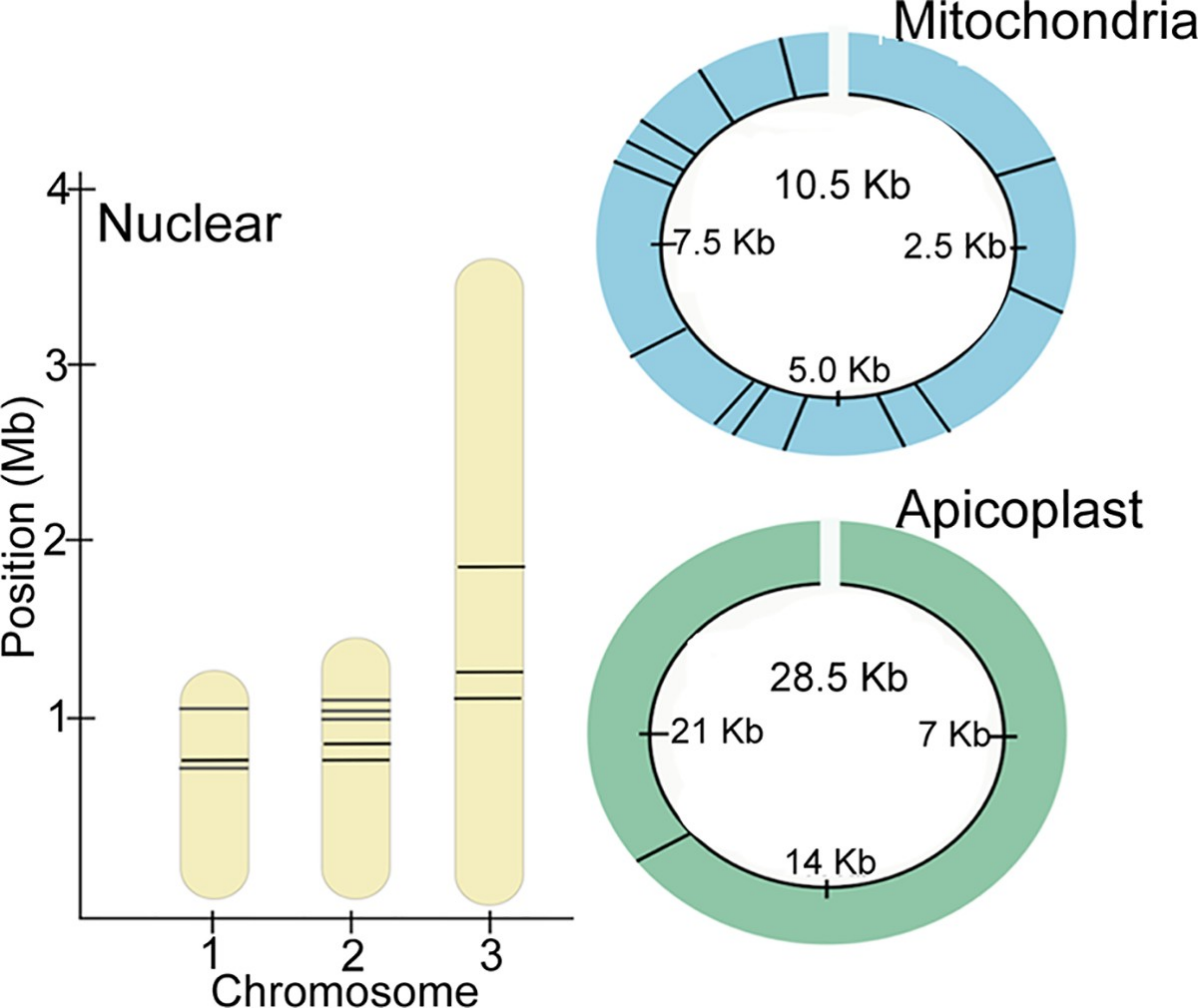
Resultant 25-SNP barcode spanning the B. microti genome. We developed our 25-SNP barcode by experimentally selecting assays that were robust and accurate. The 25 SNPs in the barcode are putatively neutral and span all nuclear chromosomes (11 SNPs), mitochondrial (13 SNPs) and apicoplastal (1 SNP) genomic regions (Fig 1). Note, the mitochondrial genome is presented as circular for illustration purposes.

**Fig 2:**
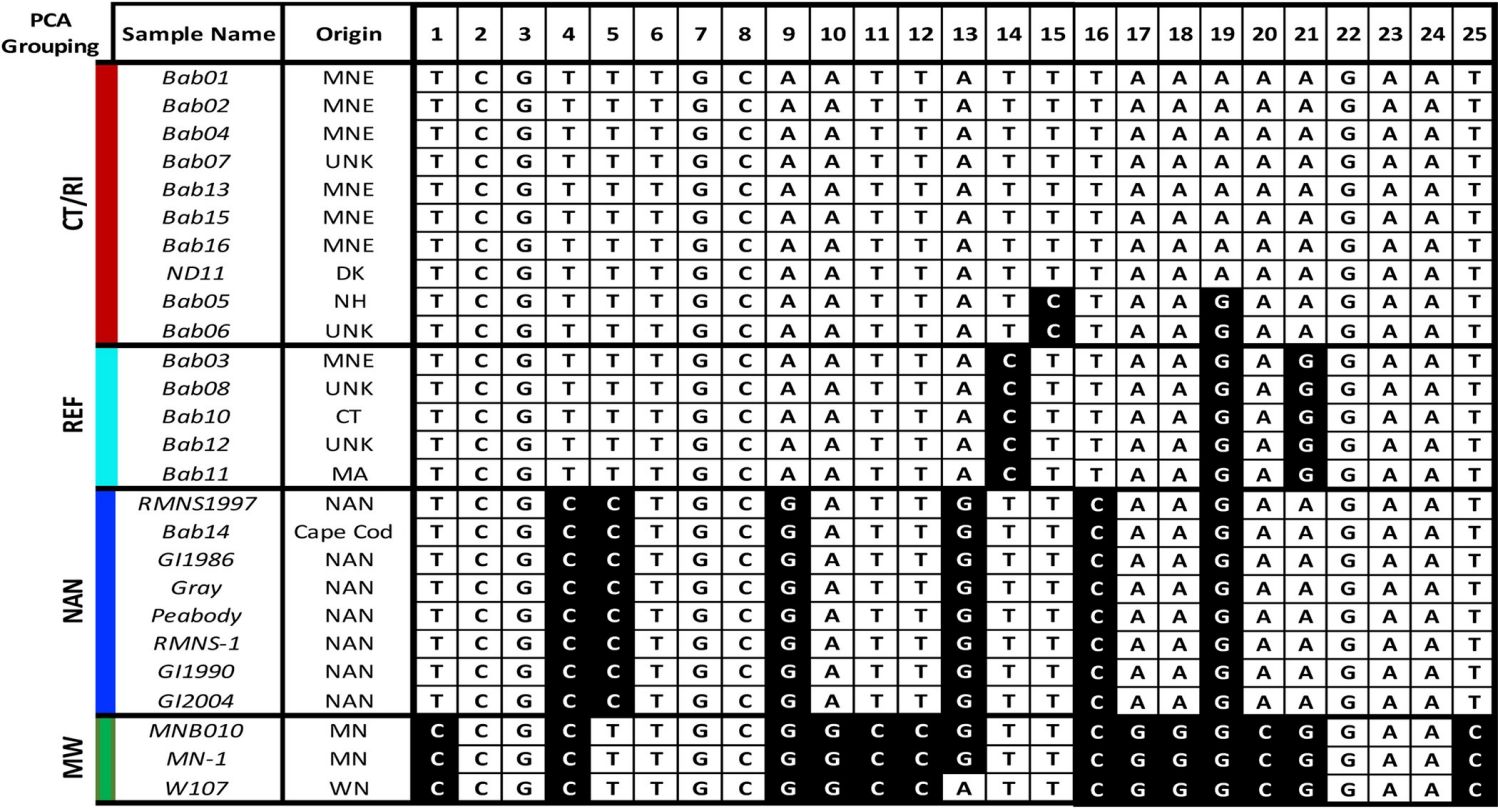
The 25-SNP barcode identifies distinct barcode signatures segregating B. microti into distinct lineages. The resultant 25-SNP barcode is shown for each sample. The top row lists the sample origin and assay number. The major allele is shown in white and the minor allele shown in black. The samples are grouped according to similar or identical barcodes and according to geographic origin Mainland New England (MNE), Reference 1 group (REF), Nantucket (NAN), and the Midwest (MW). The SNP genotyping was 100% successful (650 out of 650 SNP calls).

**Fig 3:**
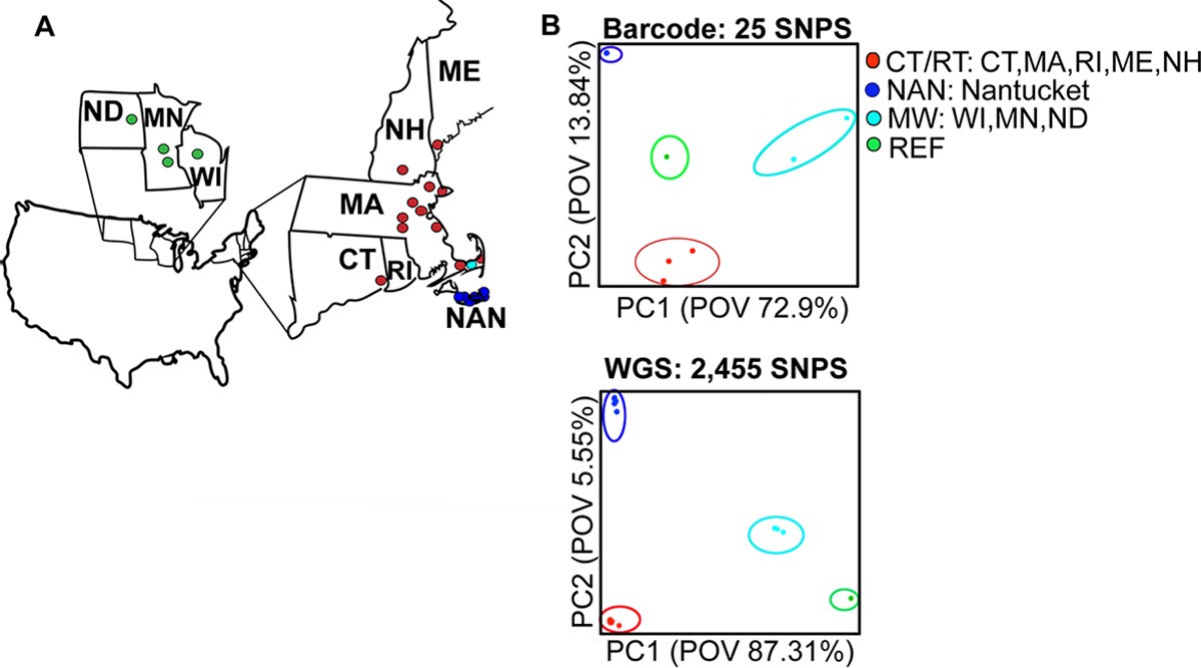
The 25-SNP barcode classifies samples by geographic origin. A) The map illustrates the four geographic origins of the samples Mainland New England (MNE—Massachusetts, Connecticut, New Hampshire, Maine, and Rhode Island) Midwest (MW—Minnesota and Wisconsin), and Nantucket with the approximate location of the sample indicated by a circle. B) The upper PCA plot shows the 25-SNP barcode separates the 26 babesiosis clinical samples into four geographic origins Mainland New England (MNE—Massachusetts, Connecticut, New Hampshire, Maine, and Rhode Island) Midwest (MW—Minnesota and Wisconsin), and Nantucket (POV = 72.92) and is comparable to lower PCA plot that shows all 2,445 SNPs (POV 87.31).

We identified a minimal barcode set creating a 25-SNP barcode maintaining nuclear (11 SNPs), mitochondrial (14 SNPs) and apicoplastal (1 SNP) regions (Fig 1). The 25-SNP barcode identified distinct barcode signatures that segregate *B*. *microti* into two lineages: NE and MW. The NE lineage has three subpopulations Connecticut/Rhode Island (CT/RI), NAN and the Reference 1 group (REF). (Fig 2). Within the lineages, the barcodes were identical or clonal, except for the MW lineage Wisconsin which differed from Minnesota by one allele (Assay 7), and the REF lineage New Hampshire which differed from Connecticut by two alleles (Assay 9 and Assay 16). The barcode captured deep divergence between CT/RI and MW lineages, with the MNE barcode consisting of 96% major alleles (24 out of 25) and the MW barcode consisting of 40% major alleles (10 out of 25 assays). Using PCA we demonstrated that the reduced 25-SNP barcode visually separated samples of different geographic origin, including CT/RI, REF, NAN, and the MW (POV = 72.92), and that it is comparable to PCA using all 2,445 SNPs (POV 87.31) (Fig 3).

## Discussion

Here, we present a *B*. *microti* 25-SNP barcode, demonstrating that it is a robust surveillance tool that could allow scientists to rapidly track and differentiate a parasites geographic origin. The increasing number of *B*. *microti* endemic regions highlights the necessity of such a surveillance tool. The 25 SNPs in the barcode are putatively neutral, with high MAF in geographically diverse regions, and span all nuclear chromosomes as well as the mitochondrial and apicoplastal genomic regions. The combination of SNPs from the nuclear, mitochondrial and apicoplastal genome leverages their distinct inheritance patterns. Apicoplastal and mitochondrial DNA SNPs help broadly differentiate geographically distinct populations, while nuclear SNPs can offer deeper insights into populations structure and genome evolution. The barcode is robust, reproducible, and sensitive, requiring only a small quantity of DNA to produce reliable results with a universal limit of detection of 10 pg. The HRM method is highly sensitive and can detect even a single nucleotide difference. We were able to successfully genotype all SNPs (468 out of 468 SNP calls) in all 26 *B*. *microti* clinical samples tested in our pilot screen.

We demonstrated the potential of the 25-SNP barcode as a surveillance tool in a pilot screen of 26 clinical samples from babesiosis endemic regions in the continental U.S.: MNE, the MW, and NAN. These 25 SNPs were able to accurately identify all samples by geographic origin, except in two cases, one from North Dakota and one from South Dennis, MA. As previously reported in our WGS study, we do not have travel information available to determine if the North Dakota case was imported case. We were, however, able to identify the South Dennis, MA case was potentially locally imported babesiosis from NAN, consistent with the barcode prediction [1]. The 25 SNPS captured high levels of clonality or minimal nucleotide diversity within the populations, and they identified unique barcode signatures that segregate *B*. *microti* into four distinct lineages: CT/RI, NAN, REF, and the MW. Further evaluation of the barcode using samples obtained from Connecticut and New Hampshire will determine if the barcode can differentiate these populations. The barcode captured deep divergence between the MNE and MW lineages.

All of our findings for the *B*. *microti* 25-SNP barcode are concordant with our WGS study [1] as well as recent genotyping studies using variable number tandem repeat (VNTR) markers [20, 21]. The 25-SNP barcode is adaptable, and scientists can further modify this barcode to fit their use. For instance, the completed 32-SNP barcode, provides 17 additional nuclear SNPs that could be evaluated in future population genetics studies. Alternatively, clinical testing the 25-SNP barcode could be further reduced to identify a minimal barcode that captures populations diversity or to create a region-specific barcode.

In summary, the *B*. *microti* 25-SNP-based barcode can serve as a baseline universal set of assays to distinguish *B*. *microti* infections based on geographic origins and to gain insight into changes in parasite population dynamics, transmission, and expansion. Our approach using HRM was strategic, the platform is flexible and can be readily updated. We envision that as research groups use this baseline set of 25-SNPs, their data and findings should be portable across studies, facilitating more accurate comparisons of results and the development of a shorter baseline set or even regional barcodes. Additionally, we envision this method to eventually evolve to potentially create SNP barcodes for additional variants, such as drug resistance, as more information about *B*. *microti* becomes available.

## Supporting information

S1 Table Detection reproducibility of the qPCR assay using 26 clinical samples. The qPCR assay was run in duplicate using 26 positive babesiosis samples and one sample positive for Lyme disease. The mean cycle threshold (CT) and standard error is reported. Samples were scored (+) for positive and (-) for negative detection of *B*. *microti*. All 26 babesiosis samples were positive and the *Lyme borreliosis* was negative by the qPCR assay.
(PDF)

S2 Table Primer sequences of the 32 HRM Assays. The forward and reverse primer sequences are listed with their corresponding optimized concentration for the HRM assay. The 25-SNP barcode is shaded in gray.
(PDF)

S3 Table Genomic properties of the 32 HRM assays. The positions of the SNPs detected by the HRM assays in the barcode are shown along with the cellular location (nuclear, mitochondrial, or apicoplastal) and corresponding Genebank ID, the reference and alternate allele for each SNP and its position. The 25-SNP barcode is shaded in gray.
(PDF)

S4 Table Efficiency and limit of detection of the 32 HRM assays. The standard curve method was performed in duplicated and the PCR efficiently and the limit of detection of both the alternate and reference allele is listed for each assay. The 25-SNP barcode is shaded in gray.
(PDF)

S5 Table Genotyping reproducibility of the 32 HRM assays. The 32 assays were screened using 26 clinical babesiosis positive samples. The assays were performed in duplicate on the Applied Biosystems ViiA 7 (384-well format). The mean Tm (°C) value and standard deviation for all SNP calls in duplicate is listed. All genotype calls were concordant across all 26 samples (832 out of 832 SNP calls) across duplicates. The 25-SNP barcode is shaded in gray.
(PDF)

S6 Table 32-SNP barcodes for the 26 clinical samples. The 32-SNP barcode was used to screen in duplicate a diverse panel of clinical samples from babesiosis endemic regions in the continental U.S.: Mainland New England (Massachusetts (8 samples), Maine (1 sample), Connecticut (1 sample), and New Hampshire (1 sample), Midwest (Wisconsin (1 sample), Minnesota (2 samples) and North Dakota (1 sample), Cape Cod (Nantucket (7 samples)), and unknow origin (4 samples). The resultant barcode is shown for each sample. The top row lists the sample origin and assay number and the major allele is shown in white and the minor allele shown in black. The 25-SNP barcode is outlined in gray. The samples are grouped according to like or identical barcodes and segregate into Mainland New England (MNE), Nantucket (NAN), R1 Reference group (REF), and Midwest (MW). The SNP genotyping was 100% (832 out of 832 SNP calls) successful.
(PDF)

S7 Table The 25-SNP barcode captures population diversity. The 25 SNP barcode captures high population diversity in babesiosis endemic regions in the continental U.S.: mainland New England (Massachusetts, Maine, Connecticut, Rhode Island and New Hampshire), Midwest (Wisconsin, Minnesota, and North Dakota), and Nantucket. The 25-SNP assay (Shaded in gray) has an average minor allele frequency (AMAF) value for each SNP > 0.22. The 32-SNP barcode has an AMAF value > 0.11 with 15 assays fixed or noninformative.
(PDF)
