# Supplementary material for: Development of a SNP barcode to genotype Babesia microti infections
Source: PLoS Negl Trop Dis. 2019 Mar 25;13(3):e0007194. doi: 10.1371/journal.pntd.0007194 (PMC6448979; doi:10.1371/journal.pntd.0007194)
Supplement: S1 Table — The qPCR assay was run in duplicate using 26 positive babesiosis samples and one sample positive for Lyme disease. The mean cycle threshold (CT) and standard error is reported. Samples were scored (+) for positive and (-) for negative detection of B. microti. All 26 babesiosis samples were positive and the Lyme borreliosis was negative by the qPCR assay. (PDF) [file pntd.0007194.s001.pdf]

Sample Name	Origin	Babesia (+/-)	CT Mean and Standard Error
Bab01	MA	+	20.92 ± 0.2
Bab02	MA	+	17.725 ± 0.23
Bab03	MA	+	15.87 ± 0.029
Bab04	MA	+	24.31 ± 0.73
Bab05	NH	+	14.32 ± 0.06
Bab06	Unknown	+	18.28 ± 0.01
Bab07	Unknown	+	18.14 ± 0.32
Bab08	Unknown	+	21.74 ± 0.08
Bab10	CT	+	17.39 ± 0.04
Bab11	MA	+	18.42 ± 0.01
Bab12	MA	+	18.07 ± 0.08
Bab13	ME	+	16.98 ± 0.04
Bab14	MA	+	19.5 ± 0.14
Bab15	MA	+	17.13 ± 0.42
Bab16	MA	+	18.75 ± 0.01
Gray	Nan	+	16.33 ± 0.18
Peabody	Nan	+	15.39 ± 0.27
RMNS-1	Nan	+	13.44 ± 0.30
GI1990	Nan	+	15.77 ± 0.09
GI1986	Nan	+	18.69 ± 0.23
GI2004	Nan	+	14.77 ± 0.11
RMNS1997	Nan	+	17.23 ± 0.02
MN-1	MI	+	18.46 ± 0.16
MNB010	MI	+	22.47 ± 0.01
ND11	ND	+	18.71 ± 0.25
W107	WI	+	22.89 ± 0.05
Lyme borreliosis	Unknown	−	−
