# Supplementary material for: Development of a SNP barcode to genotype Babesia microti infections
Source: PLoS Negl Trop Dis. 2019 Mar 25;13(3):e0007194. doi: 10.1371/journal.pntd.0007194 (PMC6448979; doi:10.1371/journal.pntd.0007194)
Supplement: S2 Table — The forward and reverse primer sequences are listed with their corresponding optimized concentration for the HRM assay. The 25-SNP barcode is shaded in gray. (PDF) [file pntd.0007194.s002.pdf]

| Barcode Assay | Forward Primer Sequence | Reverse Primer Sequence | Forward Primer (ng) | Reverse Primer (ng) |
|---|---|---|---|---|
| 1 | CTAGCTAGGCCGGAGGG | TGAAACTCGTCCAGCGT | 200 | 200 |
| 2 | GGGGTGATTTGTATTAATTTAGAGCA | ATCTCTCCAGGTAAGTTAGTGAC | 200 | 200 |
| 3 | GTACCTGCTAGCCCACTG | GAATGTTTCCCAAACCCGC | 400 | 400 |
| 4 | TCCCGTTCGACTCTCCG | CGTGCCTGTTTGATGTTG | 400 | 400 |
| 5 | ACAGTCAGCTGTGTGATG | GAGTCGTTGGTATGAAGTGG | 400 | 400 |
| 6 | TTCCTCATATGTGGTGCATATGATTAAA | AGGAACAATGTGTGTCTCG | 400 | 400 |
| 7 | GGACCACTACCTAGGGC | CTACGTCATCTACATGAAATGCT | 200 | 200 |
| 8 | TCGTGTCTACATTATATCAGAGGGT | TATTTCCCGTCTAGCCAGC | 200 | 200 |
| 9 | ACCGTATGTCTGCATTATGTTT | CAATGTCCCAGACAATAACATCC | 200 | 200 |
| 10 | ACACGGTGTTAAAACTGCAA | ATACAATTTATCCTAATGATGACGGG | 200 | 200 |
| 11 | CGCCGTAAGACCACCTC | CTCTTGGCCCTGCAGAC | 400 | 400 |
| 12 | CTACTGGGGAGCTACGG | TCCTTTGTTCCAGTAGAACAAGTTA | 200 | 200 |
| 13 | AATTACCATACATGTTTCTTTAACCCA | CTGCACTACAATATACCTACATGTTAATC | 200 | 200 |
| 14 | AGCTTGAAGTGTATAGGAATGC | ACACAGATGTTATATAGATAGACCAAGA | 200 | 200 |
| 15 | AAGAATGAAGTGTTATAAAGCTGAGT | TCATGCAGGTGTATCCTGT | 200 | 200 |
| 16 | ACTCACTGCACTACAATATACCT | AATTACCATACATGTTTCTTTAACCCA | 200 | 200 |
| 17 | CTACACATATTCTGAGGGATAGTTAG | CCAACTGTTTTAGCTAGTCTATAACTG | 200 | 200 |
| 18 | GTCCTATAGTACACCACAAGCA | AAAGGAGAATGTCTTATCCTTACAAC | 200 | 200 |
| 19 | TACTCGATAATAGGCAGTGTTATTCT | CAAGCTAATAAACAGGAGTAATTCAG | 200 | 200 |
| 20 | CATTGATACGTTAGGATTGACAG | AATATTAGCATGGGATCATTAAAGCTAT | 200 | 200 |
| 21 | CGCAGAATGCTCTTTAATGTATTT | ATCTAGTCCTGTACTTTAAGATTAATATCG | 200 | 200 |
| 22 | AGCCATCATCTCTTCATAGCTAC | TGTCGACTCATCATGGCTTAATTT | 200 | 200 |
| 23 | AGTACTAGTTATGCAGATACTAACAGG | ACAGCTACATAGTTAAATGACAGTATTAC | 200 | 200 |
| 24 | AGTCTGCATCTACATGTCTTG | CCTTACGTACTCAGGTGTTTCA | 200 | 200 |
| 25 | AGGAGGTGAACTTACAAATTTTGTTA | TCTGTTAATACACCTTCATCTAGTACTTG | 200 | 200 |
| 26 | TAAACCTGAACCAATGAGGGATAG | GCCAATCATCCGTGGCA | 400 | 400 |
| 27 | TAGGAGAATCGCTCCCTGG | TATGACCGTTACCTGTGAACC | 400 | 400 |
| 28 | CTAGCATATCCAACTGCATGTTC | TCAAGAGTCTCATTGCCTGTTA | 299 | 200 |
| 29 | GGTCAATTGTTGATGGTCAGT | GCCGAGAGACGAAAATCC | 200 | 200 |
| 30 | GTTAGCTTACTGAGCAGGTG | AAATTATATGCGTTTGTCCAAGAG | 200 | 200 |
| 31 | TATAGCTCAACTGGATGGTTTAGAT | GGGCGATAATAAAGGGTAGTG | 200 | 200 |
| 32 | AGGTTTAGTAACAATGCTAGTGTTTAT | TTGTTCCTCTGCTCCACTG | 200 | 200 |
