# Supplementary material for: Development of a SNP barcode to genotype Babesia microti infections
Source: PLoS Negl Trop Dis. 2019 Mar 25;13(3):e0007194. doi: 10.1371/journal.pntd.0007194 (PMC6448979; doi:10.1371/journal.pntd.0007194)
Supplement: S3 Table — The positions of the SNPs detected by the HRM assays in the barcode are shown along with the cellular location (nuclear, mitochondrial, or apicoplastal) and corresponding Genebank ID, the reference and alternate allele for each SNP and its position. The 25-SNP barcode is shaded in gray. (PDF) [file pntd.0007194.s003.pdf]

Barcode Assay	Cellular Location (Nuclear, Mitochondiral, Apicoplastal)	Genebank ID|reference strain|SNP Position	Amplicon (Size bp)	Reference Allele	Alternate Allele
1	nuclear (chromosome 1)	gi|399215784|emb|FO082871.1|_741112	48	C	T
2	nuclear (chromosome 1)	gi|399215784|emb|FO082871.1|_734209	57	A	C
3	nuclear (chromosome 1)	gi|399215784|emb|FO082871.1|_1279670	51	A	G
4	nuclear (chromosome 2)	gi|399216479|emb|FO082872.1|_779974	47	C	T
5	nuclear (chromosome 2)	gi|399216479|emb|FO082872.1|_823467	68	T	C
6	nuclear (chromosome 2)	gi|399216479|emb|FO082872.1|_1257020	71	C	T
7	nuclear (chromosome 2)	gi|399216479|emb|FO082872.1|_1061756	50	A	G
8	nuclear (chromosome 2)	gi|399216479|emb|FO082872.1|_1354157	67	T	C
9	nuclear (chromosome 3)	gi|399217317|emb|FO082874.1|_196486	59	G	A
10	nuclear (chromosome 3)	gi|399217317|emb|FO082874.1|_1385017	61	G	A
11	nuclear (chromosome 3)	gi|399217317|emb|FO082874.1|_1482509	45	C	T
12	mitochondrial	gi|400131493|ref|NC_018345.1|_5024	49	C	T
13	mitochondrial	gi|400131493|ref|NC_018345.1|_2404	73	G	A
14	mitochondrial	gi|400131493|ref|NC_018345.1|_3097	73	T	C
15	mitochondrial	gi|400131493|ref|NC_018345.1|_6574	50	T	C
16	mitochondrial	gi|400131493|ref|NC_018345.1|_5987	73	C	T
17	mitochondrial	gi|400131493|ref|NC_018345.1|_6322	58	G	A
18	mitochondrial	gi|400131493|ref|NC_018345.1|_8452	64	G	A
19	mitochondrial	gi|400131493|ref|NC_018345.1|_8840	54	G	A
20	mitochondrial	gi|400131493|ref|NC_018345.1|_9435	83	C	A
21	mitochondrial	gi|400131493|ref|NC_018345.1|_8598	70	G	A
22	mitochondrial	gi|400131493|ref|NC_018345.1|_10124	50	A	G
23	mitochondrial	gi|400131493|ref|NC_018345.1|_4730	57	G	A
24	mitochondrial	gi|400131493|ref|NC_018345.1|_5769	62	A	G
25	apicoplastal	gi|658131431|emb|LK028575.1|_18841	129	C	T
26	nuclear (chromosome 1)	gi|399215784|emb|FO082871.1|_1278869	63	C	A
27	nuclear (chromosome 2)	gi|399216479|emb|FO082872.1|_469723	60	A	C
31	nuclear (chromosome 2)	gi|399216479|emb|FO082872.1|_522385	48	G	A
28	nuclear (chromosome 3)	gi|399217317|emb|FO082874.1|_460856	66	G	T
29	nuclear (chromosome 3)	gi|399217317|emb|FO082874.1|_1343398	54	G	A
30	nuclear (chromosome 3)	gi|399217317|emb|FO082874.1|_1350472	69	C	T
32	mitochondrial	gi|400131493|ref|NC_018345.1|_5474	63	T	C
