# Supplementary material for: Development of a SNP barcode to genotype Babesia microti infections
Source: PLoS Negl Trop Dis. 2019 Mar 25;13(3):e0007194. doi: 10.1371/journal.pntd.0007194 (PMC6448979; doi:10.1371/journal.pntd.0007194)
Supplement: S4 Table — The standard curve method was performed in duplicated and the PCR efficiently and the limit of detection of both the alternate and reference allele is listed for each assay. The 25-SNP barcode is shaded in gray. (PDF) [file pntd.0007194.s004.pdf]

Barcode Assay	Efficiencey	r^2	Limit of Detection Total DNA (ng/µl)
1	96.38	0.992	10
2	90.07	0.993	10
3	97.79	0.994	10
4	94.78	0.996	10
5	93.74	0.998	10
6	90.80	0.999	10
7	90.63	0.997	10
8	91.78	0.999	10
9	96.91	0.984	10
10	102.33	0.908	10
11	91.33	0.999	10
12	101.54	0.993	1
13	100.25	0.997	1
14	100.57	0.974	10
15	97.57	0.952	1
16	90.70	0.990	10
17	101.29	0.957	1
18	90.92	0.997	1
19	93.22	0.997	10
20	100.93	0.994	1
21	90.08	0.993	10
22	94.89	0.999	10
23	98.82	0.996	1
24	98.46	0.988	1
25	95.29	0.995	1
26	90.13	0.991	10
27	92.92	0.993	10
28	97.66	0.993	10
29	93.55	0.991	10
30	92.26	0.992	10
31	91.44	0.978	10
32	100.12	0.942	1
