# Supplementary material for: Development of a SNP barcode to genotype Babesia microti infections
Source: PLoS Negl Trop Dis. 2019 Mar 25;13(3):e0007194. doi: 10.1371/journal.pntd.0007194 (PMC6448979; doi:10.1371/journal.pntd.0007194)
Supplement: S5 Table — The 32 assays were screened using 26 clinical babesiosis positive samples. The assays were performed in duplicate on the Applied Biosystems ViiA 7 (384-well format). The mean Tm (°C) value and standard deviation for all SNP calls in duplicate is listed. All genotype calls were concordant across all 26 samples (832 out of 832 SNP calls) across duplicates. The 25-SNP barcode is shaded in gray. (PDF) [file pntd.0007194.s005.pdf]

Barcode Assay	Tm (◦C) Reference Allele	Tm (◦C) Alternate Allele	ΔTm (◦C)
1	87.11 ± 0.10	86.26 ± 0.13	0.85
2	76.19 ± 0.02	77.19 ± 0.08	1.00
3	82.60 ± 0.09	83.55 ± 0.12	0.95
4	83.80 ± 0.11	82.89 ± 0.09	0.91
5	81.53 ± 0.09	82.49 ± 0.11	0.96
6	78.84 ± 0.15	77.99 ± 0.06	0.85
7	80.00 ± 0.08	81.00 ± 0.07	1.00
8	76.16 ± 0.15	77.28 ± 0.16	1.12
9	80.40 ± 0.15	81.30 ± 0.11	0.90
10	77.34 ± 0.12	76.40 ± 0.15	0.94
11	85.13 ± 0.15	84.20 ± 0.13	0.93
12	75.93 ± 0.12	74.97 ± 0.13	0.96
13	74.15 ± 0.06	75.05 ± 0.07	0.90
14	76.91 ± 0.11	77.73 ± 0.05	0.82
15	75.49 ± 0.14	76.46 ± 0.10	0.97
16	75.13 ± 0.09	76.00 ± 0.06	0.87
17	75.51 ± 0.09	74.45 ± 0.12	1.06
18	76.00 ± 0.06	75.21 ± 0.09	0.79
19	74.46 ± 0.13	73.26 ± 0.15	1.20
20	75.81 ± 0.12	76.64 ± 0.10	0.83
21	74.81 ± 0.10	75.66 ± 0.10	0.85
22	75.10 ± 0.14	76.07 ± 0.16	0.97
23	74.43 ± 0.15	73.52 ± 0.12	0.91
24	77.38 ± 0.09	78.30 ± 0.03	0.92
25	74.32 ± 0.18	73.44 ± 0.20	0.88
26	84.00 ± 0.04	83.47 ± 0.06	0.53
27	82.21 ± 0.07	83.15 ± 0.11	0.94
28	79.83 ± 0.04	79.00 ± 0.05	0.83
29	81.35 ± 0.14	82.30 ± 0.12	0.95
30	78.63 ± 0.05	77.69 ± 0.13	0.94
31	76.64 ± 0.15	75.75 ± 0.16	0.89
32	78.18 ± 0.16	77.38 ± 0.13	0.80
