# Supplementary material for: Development of a SNP barcode to genotype Babesia microti infections
Source: PLoS Negl Trop Dis. 2019 Mar 25;13(3):e0007194. doi: 10.1371/journal.pntd.0007194 (PMC6448979; doi:10.1371/journal.pntd.0007194)
Supplement: S6 Table — The 32-SNP barcode was used to screen in duplicate a diverse panel of clinical samples from babesiosis endemic regions in the continental U.S.: Mainland New England (Massachusetts (8 samples), Maine (1 sample), Connecticut (1 sample), and New Hampshire (1 sample), Midwest (Wisconsin (1 sample), Minnesota (2 samples) and North Dakota (1 sample), Cape Cod (Nantucket (7 samples)), and unknow origin (4 samples). The resultant barcode is shown for each sample. The top row lists the sample origin and assay number and the major allele is shown in white and the minor allele shown in black. The 25-SNP barcode is outlined in gray. The samples are grouped according to like or identical barcodes and segregate into Mainland New England (MNE), Nantucket (NAN), R1 Reference group (REF), and Midwest (MW). The SNP genotyping was 100% (832 out of 832 SNP calls) successful. (PDF) [file pntd.0007194.s006.pdf]

Sample Name	Origin	Origin	1	2	3	4	5	6	7	8	9	10	11	12	13	14	15	16	17	18	19	20	21	22	23	24	25	26	27	28	29	30	31	32
Bab01	Natick, MA	MNE	T	C	G	T	T	T	G	C	A	A	T	T	A	T	T	T	A	A	A	A	A	G	A	A	T	A	C	A	T	A	T	C
Bab02	Millis, MA	MNE	T	C	G	T	T	T	G	C	A	A	T	T	A	T	T	T	A	A	A	A	A	G	A	A	T	A	C	A	T	A	T	C
Bab04	Bedford, MA	MNE	T	C	G	T	T	T	G	C	A	A	T	T	A	T	T	T	A	A	A	A	A	G	A	A	T	A	C	A	T	A	T	C
Bab07	Unkown	UNK	T	C	G	T	T	T	G	C	A	A	T	T	A	T	T	T	A	A	A	A	A	G	A	A	T	A	C	A	T	A	T	C
Bab13	Kennebunk, ME	MNE	T	C	G	T	T	T	G	C	A	A	T	T	A	T	T	T	A	A	A	A	A	G	A	A	T	A	C	A	T	A	T	C
Bab15	Winchester, MA	MNE	T	C	G	T	T	T	G	C	A	A	T	T	A	T	T	T	A	A	A	A	A	G	A	A	T	A	C	A	T	A	T	C
Bab16	Gloucester, MA	MNE	T	C	G	T	T	T	G	C	A	A	T	T	A	T	T	T	A	A	A	A	A	G	A	A	T	A	C	A	T	A	T	C
ND11	North Dakota	DK	T	C	G	T	T	T	G	C	A	A	T	T	A	T	T	T	A	A	A	A	A	G	A	A	T	A	C	A	T	A	T	C
Bab05	Lyndeborough, NH	NH	T	C	G	T	T	T	G	C	A	A	T	T	A	T	C	T	A	A	G	A	A	G	A	A	T	A	C	A	T	A	T	C
bab06	Unkown	UNK	T	C	G	T	T	T	G	C	A	A	T	T	A	T	C	T	A	A	G	A	A	G	A	A	T	A	C	A	T	A	T	C
Bab03	Topsfield, MA	MNE	T	C	G	T	T	T	G	C	A	A	T	T	A	C	T	T	A	A	G	A	G	G	A	A	T	A	C	A	T	A	T	C
Bab08	Unkown	UNK	T	C	G	T	T	T	G	C	A	A	T	T	A	C	T	T	A	A	G	A	G	G	A	A	T	A	C	A	T	A	T	C
Bab10	Stonington, CT	CT	T	C	G	T	T	T	G	C	A	A	T	T	A	C	T	T	A	A	G	A	G	G	A	A	T	A	C	A	T	A	T	C
Bab12	Unkown	UNK	T	C	G	T	T	T	G	C	A	A	T	T	A	C	T	T	A	A	G	A	G	G	A	A	T	A	C	A	T	A	T	C
Bab11	Norwell, MA	MNE	T	C	G	T	T	T	G	C	A	A	T	T	A	C	T	T	A	A	G	A	G	G	A	A	T	A	C	A	T	A	T	C
RMNS1997	Nantucket, MA	NAN	T	C	G	C	C	T	G	C	G	A	T	T	G	T	T	C	A	A	G	A	A	G	A	A	T	A	C	A	T	A	T	C
Bab14	South Dennis, MA	Cape Cod	T	C	G	C	C	T	G	C	G	A	T	T	G	T	T	C	A	A	G	A	A	G	A	A	T	A	C	A	T	A	T	C
GI1986	Nantucket, MA	NAN	T	C	G	C	C	T	G	C	G	A	T	T	G	T	T	C	A	A	G	A	A	G	A	A	T	A	C	A	T	A	T	C
Gray	Nantucket, MA	NAN	T	C	G	C	C	T	G	C	G	A	T	T	G	T	T	C	A	A	G	A	A	G	A	A	T	A	C	A	T	A	T	C
Peabody	Nantucket, MA	NAN	T	C	G	C	C	T	G	C	G	A	T	T	G	T	T	C	A	A	G	A	A	G	A	A	T	A	C	A	T	A	T	C
RMNS-1	Nantucket, MA	NAN	T	C	G	C	C	T	G	C	G	A	T	T	G	T	T	C	A	A	G	A	A	G	A	A	T	A	C	A	T	A	T	C
GI1990	Nantucket, MA	NAN	T	C	G	C	C	T	G	C	G	A	T	T	G	T	T	C	A	A	G	A	A	G	A	A	T	A	C	A	T	A	T	C
GI2004	Nantucket, MA	NAN	T	C	G	C	C	T	G	C	G	A	T	T	G	T	T	C	A	A	G	A	A	G	A	A	T	A	C	A	T	A	T	C
MNB010	Minnesota	MN	C	C	G	C	T	T	G	C	G	G	C	C	G	T	T	C	G	G	G	C	G	G	A	A	C	A	C	A	T	A	T	C
MN-1	Minnesota	MN	C	C	G	C	T	T	G	C	G	G	C	C	G	T	T	C	G	G	G	C	G	G	A	A	C	A	C	A	T	A	T	C
W107	Wisconsin	WN	C	C	G	C	T	T	G	C	G	G	C	C	A	T	T	C	G	G	G	C	G	G	A	A	C	A	C	A	T	A	T	C
