# Supplementary material for: Development of a SNP barcode to genotype Babesia microti infections
Source: PLoS Negl Trop Dis. 2019 Mar 25;13(3):e0007194. doi: 10.1371/journal.pntd.0007194 (PMC6448979; doi:10.1371/journal.pntd.0007194)
Supplement: S7 Table — The 25 SNP barcode captures high population diversity in babesiosis endemic regions in the continental U.S.: mainland New England (Massachusetts, Maine, Connecticut, Rhode Island and New Hampshire), Midwest (Wisconsin, Minnesota, and North Dakota), and Nantucket. The 25-SNP assay (Shaded in gray) has an average minor allele frequency (AMAF) value for each SNP > 0.22. The 32-SNP barcode has an AMAF value > 0.11 with 15 assays fixed or noninformative. (PDF) [file pntd.0007194.s007.pdf]

Barcode Assay	Major Allele	Minor Allele	Minor Allel frequency (MAF)
1	T	C	0.12
2	C	A	0.00
3	G	A	0.00
4	T	C	0.42
5	T	C	0.31
6	T	C	0.00
7	G	A	0.00
8	C	T	0.00
9	A	G	0.42
10	A	G	0.10
11	T	C	0.10
12	T	C	0.10
13	A	G	0.38
14	T	C	0.19
15	T	C	0.08
16	T	C	0.40
17	A	G	0.10
18	A	G	0.10
19	G	A	0.31
20	A	G	0.10
21	A	G	0.31
22	G	A	0.00
23	A	G	0.00
24	A	G	0.00
25	T	C	0.10
26	A	C	0.00
27	C	A	0.00
28	A	G	0.00
29	T	G	0.00
30	A	G	0.00
31	T	C	0.00
32	C	T	0.00
